# Using multi-omics to explore the genetic causal relationship between colorectal cancer and heart failure in gastrointestinal tumors

**DOI:** 10.3389/fimmu.2024.1454021

**Published:** 2024-09-13

**Authors:** Hongjing Han, Xuefang Cai, Xiangling Liu

**Affiliations:** ^1^ Section 2 of General Surgery Department, The Second People’s Hospital of Jingdezhen, Jingdezhen, China; ^2^ Hemodialysis Department, The Second People’s Hospital of Jingdezhen, Jingdezhen, China

**Keywords:** heart failure, colorectal cancer, Mendelian randomization, causal relationship, GWAS

## Abstract

**Background:**

Heart failure (HF) and colorectal cancer are significant public health concerns with substantial morbidity and mortality. Previous studies have indicated a close association between HF and various tumors, including colorectal cancer. Further understanding the potential causal relationship between them could provide insights into their shared pathophysiological mechanisms and inform strategies for prevention and treatment.

**Methods:**

This study employed a bidirectional Mendelian randomization (MR) approach using genetic variants from large genome-wide association studies (GWAS) as instrumental variables (IVs). The inverse-variance weighted (IVW) method was employed for the MR analysis. Meta-analyses of IVW results from discovery and validation cohorts were performed to enhance the power of detecting causal effects. Sensitivity analyses, including heterogeneity analysis and tests for horizontal pleiotropy, were conducted to test the robustness of the conclusions.

**Results:**

Results from the discovery cohort suggest HF is associated with an approximately 30% increased risk of colorectal cancer (OR 1.32, 95% CI 1.03-1.69, P=0.025), although this finding did not reach statistical significance in the validation cohort (OR 1.19, 95% CI 0.97-1.46, P=0.090). However, meta-analysis supports HF as a potential risk factor for colorectal cancer (Pooled OR 1.24, 95% CI 1.06-1.25, P=0.007). Reverse MR analysis found no evidence of colorectal cancer increasing HF risk (Pooled OR 1.03, 95% CI 0.99-1.07, P=0.121). Sensitivity analyses (all P>0.05) indicate robustness against heterogeneity and horizontal pleiotropy.

**Conclusion:**

This comprehensive bidirectional MR study provides genetic evidence supporting a causal link between HF and colorectal cancer. The insights gained enhance understanding of their interconnectedness and may guide future research and clinical practices aimed at mitigating their risks through targeted interventions.

## Introduction

Heart failure (HF) is a clinical syndrome characterized by inadequate pumping function of the heart, affecting tens of millions of individuals worldwide (1–4). As a multifactorial disease, HF is closely associated with cardiovascular risk factors such as aging, hypertension, and diabetes (5–7). Patients with HF often exhibit symptoms such as dyspnea and reduced exercise tolerance (8–10), significantly impacting their quality of life and survival (11, 12). Therefore, in-depth research on HF is not only crucial for improving patient outcomes but also essential for reducing overall healthcare burden (13, 14).

Several studies have investigated the relationship between HF and various tumors, suggesting potential shared pathophysiological mechanisms or treatment interactions (15–17). For instance, certain medications used for HF treatment may influence tumor growth and development, and vice versa (18, 19). Additionally, chronic inflammatory states play a significant role in the onset of both HF and tumors (20). Colorectal cancer is one of the tumor types that deserve special attention in this context, as its incidence continues to rise globally and is closely linked to genetic factors, dietary habits, and lifestyle (21, 22). A previous cross-sectional study suggested that HF may be a potential risk factor for colorectal cancer (17). For instance, one study found an association between HF and an increased risk of various cancers, including CRC, highlighting the need for further investigation into the underlying mechanisms and potential causal relationships. Similarly, another study observed an increased incidence of cancer in HF patients but emphasized that observational studies are prone to confounding factors such as environmental influences and comorbidities. To address these gaps, this study employs Mendelian randomization (MR) to explore the bidirectional causal link between HF and CRC. MR utilizes genetic variants as instrumental variables (IVs) to investigate causal relationships, leveraging the random allocation of genetic variations to mitigate confounding biases inherent in traditional observational studies. This method provides more precise evidence for understanding the underlying biological mechanisms of diseases. For example, one MR study provided evidence supporting a causal relationship between elevated body mass index (BMI), a known risk factor for both HF and CRC, and an increased risk of these conditions. Another MR study demonstrated that genetic predisposition to higher BMI and inflammation is associated with an increased risk of HF and CRC, suggesting common pathophysiological pathways. Considering that observational studies are highly susceptible to confounding factors such as environmental influences, we further explored the bidirectional causal link between HF and colorectal cancer through Mendelian randomization (MR) study (23). MR studies represent a powerful tool that utilizes genetic variants as instrumental variables (IVs) to investigate the causal relationship between HF and colorectal cancer. This innovative method in epidemiology leverages the random allocation of genetic variations, effectively mitigating confounding biases inherent in traditional observational studies (24, 25). As a result, MR provides more precise evidence for understanding the underlying biological mechanisms of diseases. In summary, the application of MR not only enhances our comprehension of the causal pathways linking these diseases but also lays a robust scientific foundation for developing targeted preventive and therapeutic strategies in the future.

## Materials and methods

### GWAS summary data source

The genome-wide association study (GWAS) data for the HF (discovery cohort) and colorectal cancer were sourced from IEU OpenGWAS (26), a publicly accessible website for researchers to obtain GWAS data, with the IDs of “ebi-a-GCST009541” (N= 977,323) and “ebi-a-GCST012879” (N=32,072), respectively. The GWAS summary data for the HF (validation cohort, N=387,444) was derived from FinnGen (27), which is a large-scale genome project aiming to explore the relationship between genes and diseases by integrating multiple healthcare databases and biobanks within Finland. The detailed information of all GWAS data used in this MR study is summarized in **Table 1**.

**Table 1:** Detailed information on GWAS for heart failure and colorectal cancer.

Traits	ID of GWAS	Year	Sample Size	Population
Heart failure (Discovery cohort)	ebi-a-GCST009541	2020	977,323	European
Heart failure (Validation cohort)	finngen_R10_I9_HEARTFAIL_EXMORE	2023	387,444	European
Colorectal Cancer	ebi-a-GCST012879	2018	32,072	European

### Selection IVs

**Figure 1:**
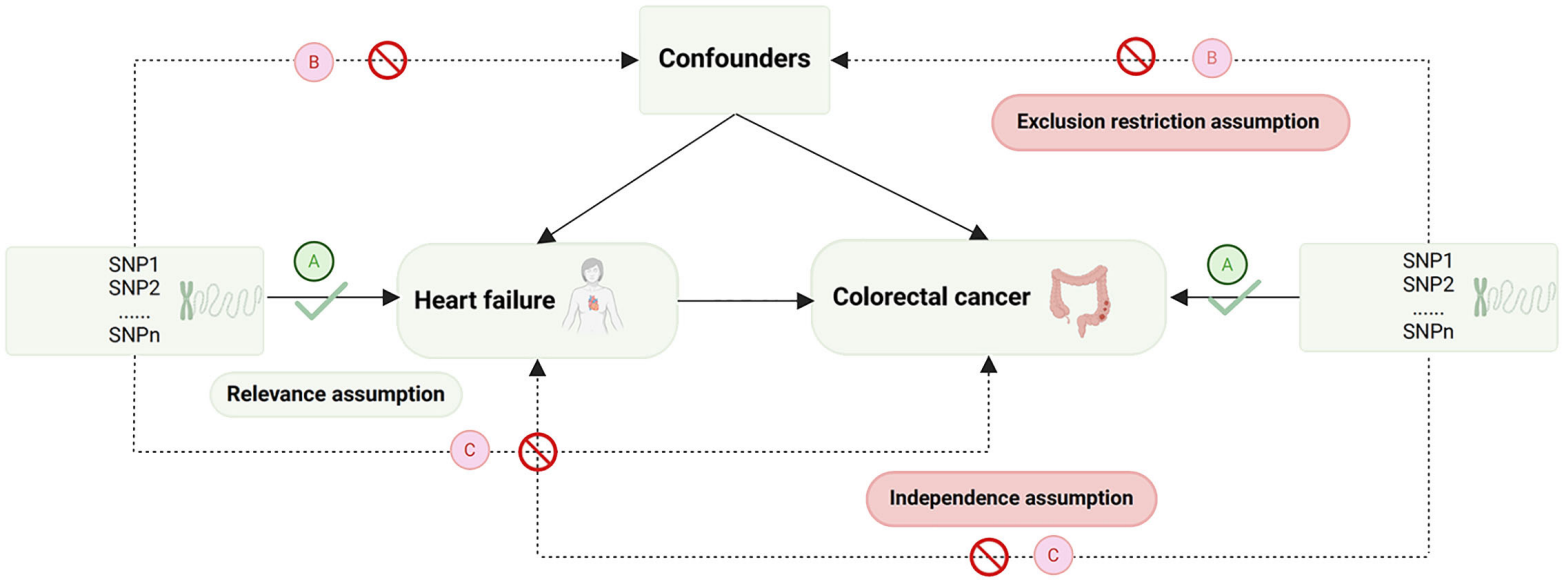
Diagram of MR basic assumptions. SNP, single nucleotide polymorphisms.

**Figure 2:**
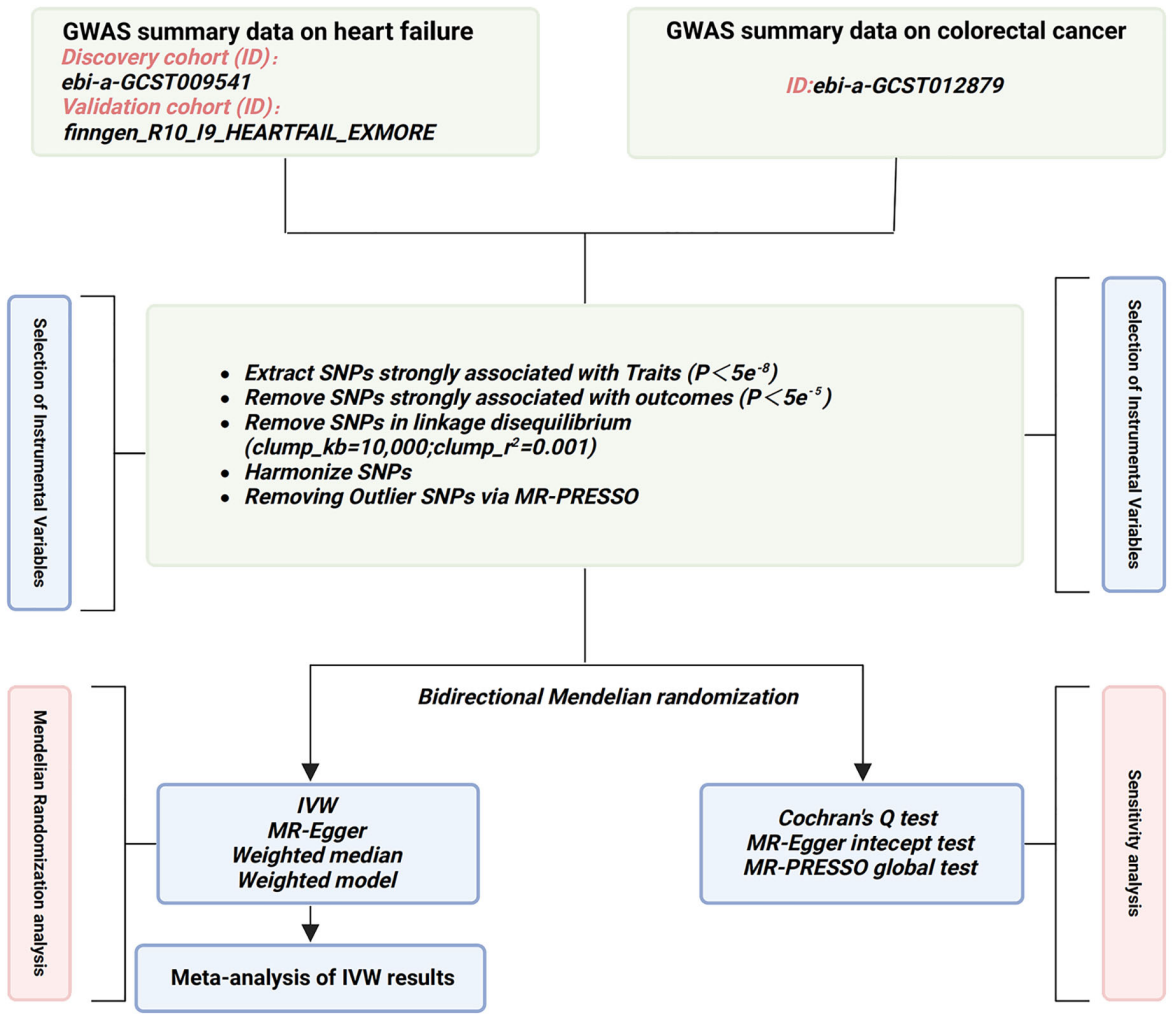
Flowchart of the study design in this MR study. GWAS, genome-wide association study; SNP, single nucleotide polymorphisms; MR-PRESSO, Mendelian randomization pleiotropy residual sum and outlier; IVW, inverse-variance weighted.

The selection of IVs is based on the fundamental assumptions of MR (28) (**Figure 1**). Initially, single nucleotide polymorphisms (SNPs) strongly associated with the exposure were extracted (*p*<5e^-8^), and those showing a robust correlation with the outcome were omitted (*p*<5e^-5^). Next, SNPs without linkage disequilibrium with other variants were selected (r^2 =^ 0.001, Kb=10,000). Subsequently, SNPs that could not harmonize with the outcome GWAS data and exhibit palindromic sequences were been eliminated. To mitigate the influence of weak IVs on the study results, only SNPs with an F-statistic>10 were chosen as the valid IVs for the subsequent MR analysis. (The F-statistic is calculated as follows: F = β^2^/se^2^) (29). Finally, outlier SNPs, identified by Mendelian randomization pleiotropy residual sum and outlier (MR-PRESSO) method (30), were removed (**Figure 2**).

### MR and statistical methods

We employed the inverse variance weighted method (IVW) (31) to assess the causal relationship between HF and colorectal cancer. Complementary analyses using MR-Egger regression, weighted median, and weighted model were conducted to support the IVW results. A meta-analysis was performed to evaluate the combined effect size based on the IVW findings. Cochran’s Q test was utilized to assess statistical heterogeneity among SNPs, while MR-Egger intercept testing and MR-PRESSO global test were employed to examine horizontal pleiotropy. A P-value > 0.05 indicated no significant heterogeneity or pleiotropy among SNPs. Leave-one-out analysis was conducted to assess the influence of individual SNPs. Statistical analyses were conducted using the “TwoSampleMR”, “MR-PRESSO”, and “meta” packages in R software. A significance threshold of P < 0.05 was applied.

### Quantitative real-time PCR analysis

The CRC cell lines (LoVo and SW480) and normal colonic epithelial cell line (NCM460) were obtained from Shanghai Zhong Qiao Xin zhou Biotechnology Co., Ltd. and ATCC (Manassas, VA, USA), respectively. These cell lines were cultured in DMEM medium (Solarbio, Beijing, China) supplemented with 10% FBS and 1% penicillin-streptomycin. Total RNA was extracted using TRIzol reagent (Invitrogen, Carlsbad, CA, USA), and RNA was reverse transcribed into cDNA using the ReverTra Ace qPCR RT Master Mix with gDNA Remover kit. Quantitative real-time PCR (qRT-PCR) was performed using SYBR Premix Ex Taq II on an Mx3005P real-time PCR system (Stratagene, San Diego, CA, USA), with GAPDH serving as the internal control for mRNA. The reaction conditions were as follows: initial denaturation at 95°C for 10 minutes, followed by 45 cycles of 95°C for 5 seconds and 60°C for 30 seconds. Each sample was amplified in triplicate for both the target gene and the internal control gene. Data analysis was performed using the 2^(-ΔΔCt) method. The primer sequences are provided in **Supplementary Table 1**.

## Results

### The causal effect of HF on colorectal cancer

**Figure 3:**
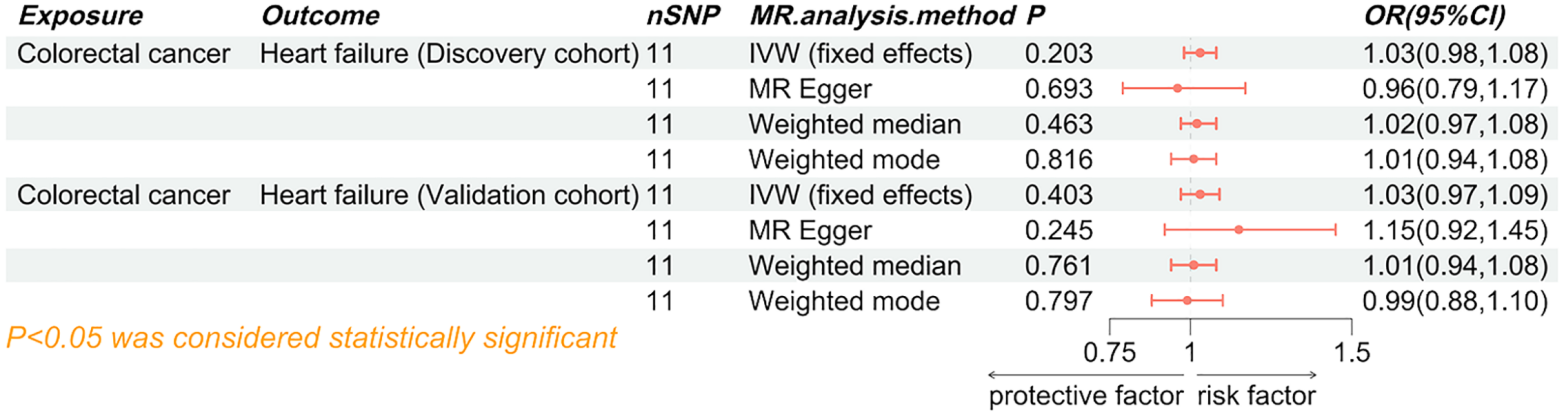
Forest plot of the causal effect of heart failure on colorectal cancer. SNP, single nucleotide polymorphisms; MR, Mendelian randomization; OR, odd ratio; 95% CI, 95% confidence interval; IVW, inverse-variance weighted.

**Figure 4:**
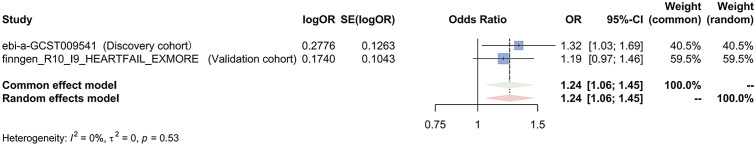
Meta-analysis of the IVW results assessing the causal effect of heart failure on colorectal cancer. OR, odd ratio; 95% CI, 95%.

The results of the IVW analysis from the discovery cohort suggest that HF is associated with an approximately 30% increased risk of colorectal cancer (odds ratio (OR) 1.32, 95% confidence interval (CI) 1.03-1.69, P=0.025). However, this finding did not reach statistical significance in the validation cohort (OR 1.19, 95% CI 0.97-1.46, P=0.090) (**Figure 3**). Nevertheless, the meta-analysis results consistently support HF as a potential risk factor for colorectal cancer (Pooled OR 1.24, 95% CI 1.06-1.25, P=0.007) (**Figure 4**). While not all additional MR methods achieved statistical significance, their OR values consistently indicated an odds ratio greater than 1. The results of a Mendelian Randomization (MR) analysis illustrate the causal relationship between a specific exposure and disease incidence (**Supplementary Figure 1**). The scatter plot shows the association between genetic variants (instrumental variables) and the exposure, with each point representing a single nucleotide polymorphism (SNP). The forest plot summarizes effect estimates from individual SNPs, with horizontal lines indicating 95% confidence intervals. The funnel plot assesses potential directional pleiotropy by displaying the inverse standard error of the SNP-outcome association against the ratio estimate of each SNP. Sensitivity analyses, including MR-Egger regression and weighted median methods, compare with the primary MR analysis. This comprehensive representation demonstrates the robustness and consistency of the causal inference from the genetic data, with statistical significance and heterogeneity measures providing additional validation for the findings.

### The causal effect of colorectal cancer on HF

**Figure 5:**
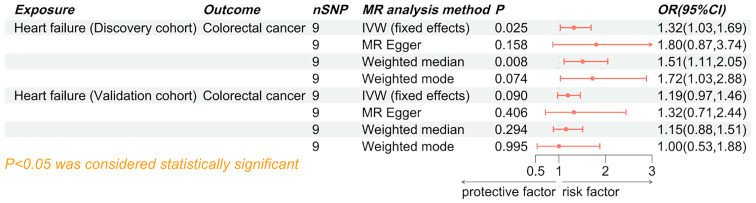
Forest plot of the causal effect of colorectal cancer on heart failure. SNP, single nucleotide polymorphisms; MR, Mendelian randomization; OR, odd ratio; 95% CI, 95% confidence interval; IVW, inverse-variance weighted.

**Figure 6:**
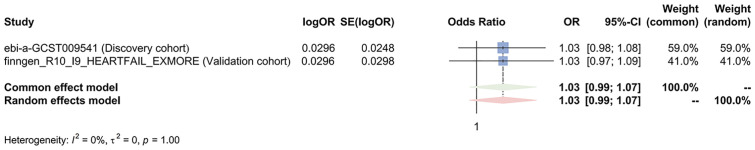
Meta-analysis of the IVW results assessing the causal effect of colorectal cancer on heart failure. OR, odd ratio; 95% CI, 95% confidence interval.

Both the discovery cohort (OR 1.03, 95% CI 0.98-1.08, P_IVW_=0.203) and validation cohort (OR 1.03, 95% CI 0.97-1.09, P_IVW_=0.403) did not indicate an increased risk of HF due to colorectal cancer (**Figure 5**). The meta-analysis of IVW results further rejected the reverse causality Pooled OR 1.03, 95% CI 0.99-1.07, P=0.121) (**Figure 6**).

MR analysis was re-conducted after removing outlier SNPs. Cochran’s Q test did not detect heterogeneity in the bidirectional MR analysis (P>0.05). the MR-Egger intercept test and MR-PRESSO global test indicated the absence of horizontal pleiotropy (P>0.05). The leave-one-out analysis suggested that the results were relatively robust (**Figure 6**). The sensitivity analysis investigates the robustness of MR study examining the causal relationship between HF and CRC (**Table 2**). The analysis evaluates heterogeneity and pleiotropy across both discovery and validation cohorts. For the discovery cohort, the heterogeneity test, indicated by Cochran’s Q test (P-value of IVW), shows a P-value of 0.271, suggesting no significant heterogeneity among the instrumental variables. The Egger intercept test for pleiotropy yields a P-value of 0.406, while the MR-PRESSO global test returns a P-value of 0.263, both indicating no significant evidence of pleiotropy. In the validation cohort, the heterogeneity test shows a P-value of 0.255, similar to the discovery cohort, suggesting consistent results with no significant heterogeneity. The Egger intercept test provides a P-value of 0.738, and the MR-PRESSO global test shows a P-value of 0.215, indicating no significant pleiotropy. The combined analysis of heart failure as the exposure in both cohorts reveals a heterogeneity test P-value of 0.181 for the discovery cohort and 0.136 for the validation cohort, suggesting no significant heterogeneity in either case. The Egger intercept test P-values are 0.479 and 0.317, respectively, and the MR-PRESSO global test P-values are 0.219 and 0.155, indicating no significant pleiotropy across both cohorts. Overall, the sensitivity analysis supports the robustness of the MR findings, with no significant heterogeneity or pleiotropy detected in either cohort, thus reinforcing the reliability of the observed genetic causal relationship between heart failure and colorectal cancer.

**Table 2:** Sensitivity analysis of this MR study.

Exposure	Outcome	Heterogeneity test	Pleiotropy test	
		Cochran’s Q test *(P-value of IVW)*	Egger intercept test *(P-value)*	MR-PRESSO global test *(P-value)*
Heart failure (Discovery cohort)	Heart failure	0.271	0.406	0.263
Heart failure (Validation cohort)	Heart failure	0.255	0.738	0.215
Heart failure	Heart failure (Discovery cohort)	0.181	0.479	0.219
Heart failure	Heart failure (Validation cohort)	0.136	0.317	0.155

### Comprehensive analysis of SLC22A3 expression and its clinical correlations in colorectal cancer

**Figure 7:**
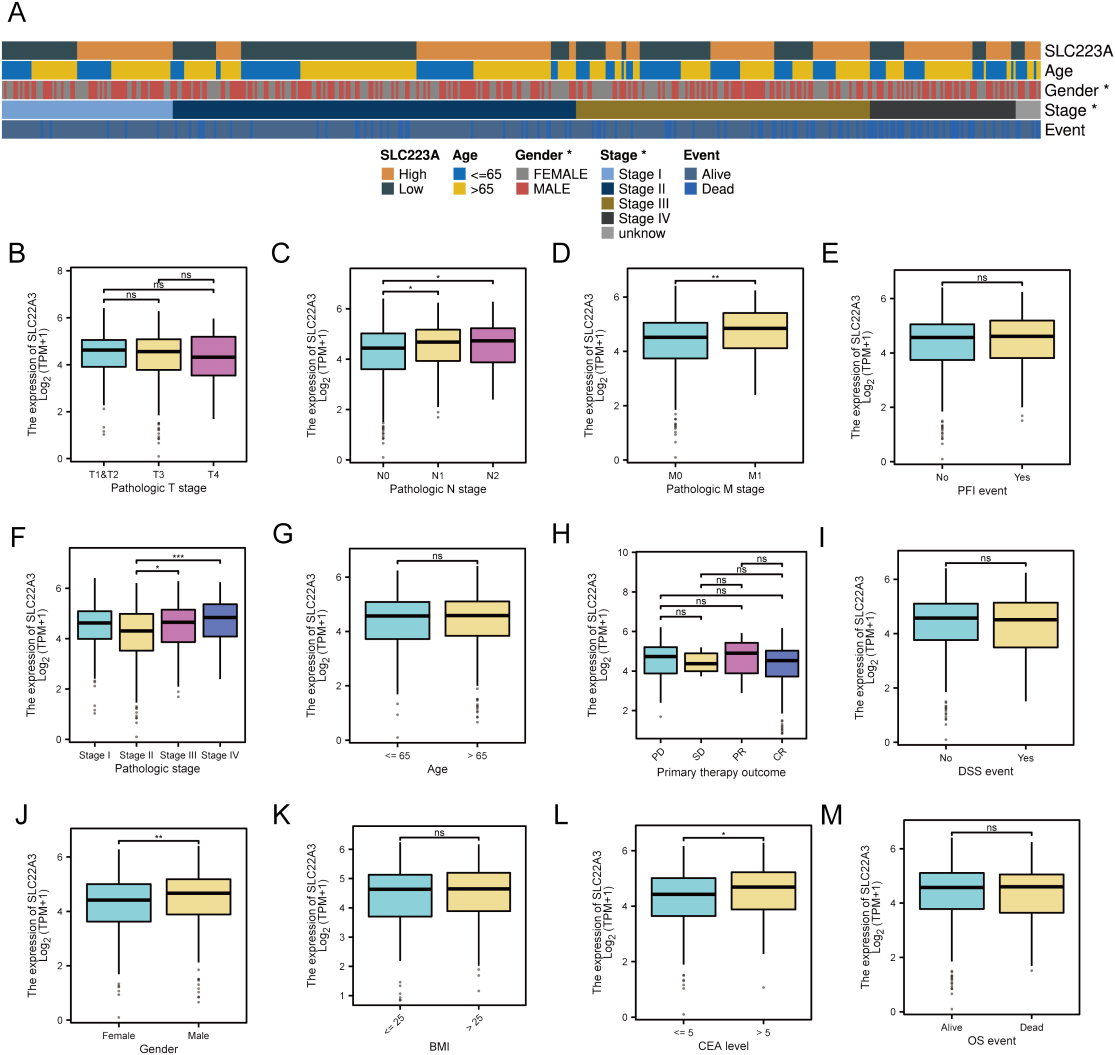
Differential expression and clinical significance of SLC22A3 in colorectal cancer samples **(A)** Correlation of SLC22A3 expression with clinical characteristics in colorectal cancer. SLC22A3 expression: Samples are categorized into high (orange) and low (dark green) expression groups. Age: Patients are divided into two age groups: ≤65 years (blue) and >65 years (yellow). Gender: Gender is indicated with female (gray) and male (red). Stage: The pathological stages of cancer are represented as follows: Stage I (light blue), Stage II (dark blue), Stage III (brown), Stage IV (black), Unknown stage (gray). Event: The survival event status of patients is shown with alive (light blue) and dead (dark blue). **(B)** SLC22A3 expression across pathologic T stages in colorectal cancer. **(C)** SLC22A3 expression Across pathologic N stages in colorectal cancer. **(D)** SLC22A3 expression across pathologic M stages in colorectal cancer. **(E)** SLC22A3 expression and progression-free interval (PFI) events in colorectal cancer. **(F)** SLC22A3 expression across different pathologic stages in colorectal cancer. **(G)** SLC22A3 expression across different age groups in colorectal cancer. **(H)** SLC22A3 expression and primary therapy outcome in colorectal cancer. **(I)** SLC22A3 expression and disease-specific survival (DSS) events in colorectal cancer. **(J)** SLC22A3 expression across Gender in colorectal cancer. **(K)** SLC22A3 expression across different BMI categories in colorectal cancer. **(L)** SLC22A3 expression across different CEA levels in colorectal cancer. **(M)** SLC22A3 expression and overall survival (OS) events in colorectal cancer. “*”: p < 0.05 (statistically significant); “**”: p < 0.01 (more significant); “***”: p < 0.001 (highly significant); “ns”: Not significant.

In this study, we investigated the expression profiles of various clinical traits and their correlation with gene expression in colorectal cancer (CRC) samples from The Cancer Genome Atlas (TCGA) dataset (**Figure 7**). The analysis segregated the samples into high and low expression groups, highlighting significant differences in gene expression patterns associated with distinct clinical characteristics. The heatmap analysis showed that SLC22A3 expression correlated with various clinical features such as age, gender, pathological stage, and survival events. Among the differentially expressed genes, several candidates stood out, including SLC22A3, CDKN1A, CELSR2, and ABO. The selection process involved a meticulous comparison of the log2 fold change and adjusted p-values (padj) of these genes. SLC22A3 exhibited significant differences in expression between pathological T and N stages, suggesting its involvement in tumor progression. Higher expression of SLC22A3 was observed in patients with distant metastasis, indicating its potential role in metastasis. However, no significant differences were seen in relation to progression-free interval (PFI) events, suggesting it may not correlate directly with progression-free survival. Significant differences in SLC22A3 expression were noted across different pathological stages but not age groups. In terms of primary therapy outcomes, no significant differences were observed, suggesting that SLC22A3 expression may not be directly correlated with therapy response. Similarly, no significant difference was seen in SLC22A3 expression between patients with and without disease-specific survival (DSS) events. Gender analysis revealed significant differences, with males showing higher SLC22A3 expression than females, but no significant differences were observed across different BMI categories. Higher SLC22A3 expression was also observed in patients with elevated carcinoembryonic antigen (CEA) levels, suggesting its association with tumor burden. However, no significant difference was found between patients who were alive and those who had died, indicating SLC22A3 may not correlate directly with overall survival. SLC22A3 exhibited a substantial log2 fold change of 1.935795, coupled with an exceptionally significant adjusted p-value (padj = 9.574958e-42), indicating its marked upregulation in CRC tissues compared to normal tissues. Similarly, CELSR2 showed a log2 fold change of 0.879212 with a padj of 8.021050e-13, suggesting its involvement in CRC. CDKN1A and ABO, though showing negative log2 fold changes, also demonstrated significant differential expression, with CDKN1A at -1.176013 (padj = 6.889234e-18) and ABO at -0.895181 (padj = 2.232410e-08).

The significant differential expressions of these genes suggest their potential roles in the pathogenesis of colorectal cancer. However, among these, SLC22A3 was chosen as the final single-gene candidate for further validation and functional studies due to its particularly strong differential expression and potential biological relevance. The identification and subsequent investigation of SLC22A3, along with other differentially expressed genes, could provide valuable insights into the molecular mechanisms underlying CRC and contribute to the development of targeted therapeutic strategies.

### Prognostic value of SLC22A3 in colorectal cancer: a comprehensive analysis

**Figure 8:**
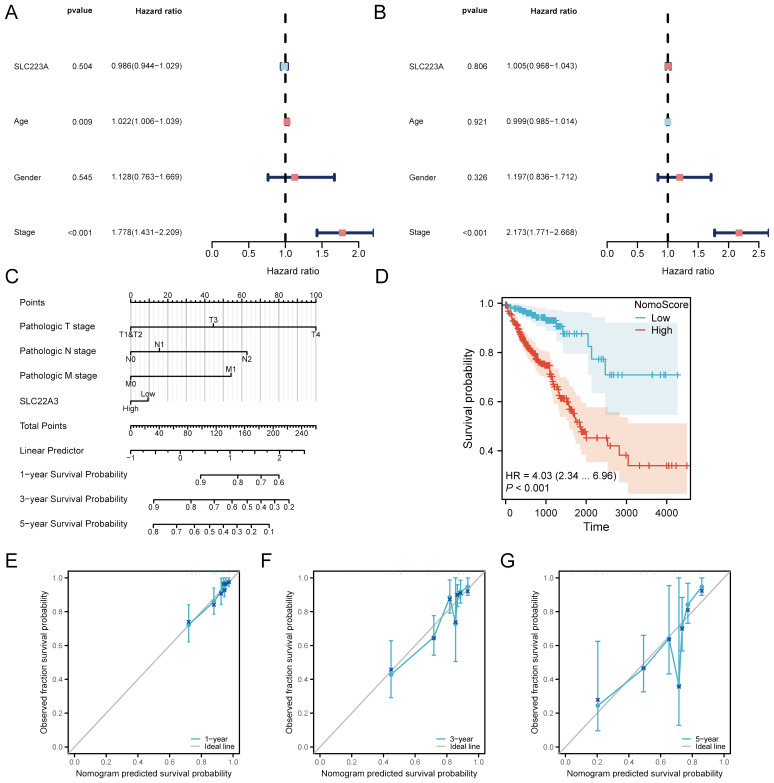
Prognostic analysis of SLC22A3 in colorectal cancer. **(A)** Univariate cox regression forest plot for overall survival (OS). **(B)** Univariate cox regression forest plot for progression-free interval (PFI). **(C)** Nomogram integrating clinical traits with SLC22A3 expression. **(D)** Kaplan-Meier survival curve based on nomogram risk score. **(E)** Calibration curve for 1-year survival probability. **(F)** Calibration curve for 3-year survival probability. **(G)** Calibration curve for 5-year survival probability.

This analysis presents a detailed examination of the prognostic value of SLC22A3 in colorectal cancer (CRC), using various statistical and visual tools to validate its clinical significance. Univariate Cox regression analysis assesses the impact of SLC22A3 expression on overall survival (OS) and progression-free interval (PFI). The forest plots from this analysis (**Figures 8A, B**) illustrate the hazard ratios (HR) and confidence intervals (CI) for SLC22A3, showing that higher expression is associated with an increased risk of poor outcomes in both OS and PFI. A nomogram integrates SLC22A3 expression with various clinical traits, including pathological T stage, N stage, and M stage, to predict individual patient outcomes. This nomogram (**Figure 8C**) provides a visual tool for estimating the probability of 1-year, 3-year, and 5-year survival rates based on cumulative scores derived from multiple prognostic factors, such as SLC22A3 expression, age, gender, and tumor stage. This comprehensive approach allows clinicians to make more informed decisions regarding patient prognosis and treatment strategies. The nomogram-derived risk score is further validated through Kaplan-Meier survival analysis (**Figure 8D**), where patients are stratified into high-risk and low-risk groups. The Kaplan-Meier curves demonstrate a significant difference in survival probabilities between these groups, with the high-risk group showing substantially poorer survival outcomes. This stratification underscores the utility of the nomogram in identifying patients with different prognostic risks. Three calibration curves (**Figures 8E–G**) assess the accuracy of the nomogram in predicting 1-year, 3-year, and 5-year survival probabilities. These calibration plots compare the predicted survival rates with the actual observed outcomes, and a close alignment along the 45-degree line indicates a high degree of predictive accuracy. This validation step ensures that the nomogram is reliable and can be effectively used in clinical settings. Overall, this comprehensive analysis underscores the significance of SLC22A3 as a prognostic biomarker in colorectal cancer, providing robust statistical evidence and practical tools for its application in personalized patient management. By integrating gene expression data with clinical traits, the findings offer valuable insights into the molecular mechanisms underlying CRC and contribute to the development of targeted therapeutic strategies.

### Prognostic significance of SLC22A3 in colorectal cancer

This comprehensive evaluation presents the prognostic significance of SLC22A3 expression in colorectal cancer (CRC), integrating risk factor analysis, receiver operating characteristic (ROC) curves, and decision curve analysis (DCA) (**Supplementary Figure 2A**). The risk factor plot illustrates the hazard ratios (HR) and confidence intervals (CI) for various clinical and molecular factors, including SLC22A3 expression, age, gender, and tumor stage. This visualization underscores the relative importance of each factor in predicting patient outcomes, with higher HR values indicating a greater associated risk. Notably, SLC22A3, along with other significant clinical parameters, demonstrates a strong correlation with adverse prognosis. The ROC curves assess the predictive accuracy of the nomogram incorporating SLC22A3 expression for 1-year, 3-year, and 5-year survival rates in CRC patients. The ROC curve for 1-year survival prediction shows an area under the curve (AUC) of 0.745, indicating a good level of discrimination (**Supplementary Figure 2E**). Similarly, **Supplementary Figure 2F** presents the ROC curve for 3-year survival prediction, also with an AUC of 0.745, demonstrating consistent predictive performance over a longer time frame. **Supplementary Figure 2G** depicts the ROC curve for 5-year survival prediction, with an AUC of 0.727, reflecting a good level of discrimination over an extended period. These curves quantify the nomogram’s discriminatory power to differentiate between patients with varying survival outcomes, with high AUC values signifying superior predictive accuracy and validating the nomogram’s robustness in forecasting survival probabilities across multiple time points. The decision curve analysis (DCA) evaluates the clinical utility of the nomogram by calculating the net benefits across a spectrum of threshold probabilities. The result shows the DCA for 1-year survival prediction, indicating higher net benefits across various threshold probabilities, thus highlighting the practical value of the nomogram in clinical decision-making (**Supplementary Figure 2B**). The DCA for 3-year survival prediction confirms that the combined risk score offers significant net benefits compared to default strategies of universal treatment versus no treatment (**Supplementary Figure 2C**). The DCA for 5-year survival prediction shows that the nomogram consistently provides higher net benefits across multiple threshold probabilities, underscoring its significant advantage in guiding long-term therapeutic decisions (**Supplementary Figure 2D**).

In summary, this thorough evaluation of SLC22A3 as a prognostic biomarker in colorectal cancer, integrating risk factor analysis, ROC curves, and DCA, establishes a robust framework for assessing the predictive accuracy and clinical relevance of the nomogram. This approach reinforces its potential utility in personalized patient management and the development of targeted therapeutic strategies. The detailed analysis, combining risk score distribution and survival correlation with SLC22A3 expression, further solidifies its importance in CRC prognosis.

### Comprehensive functional enrichment analysis of SLC22A3 expression in colorectal cancer

**Figure 9:**
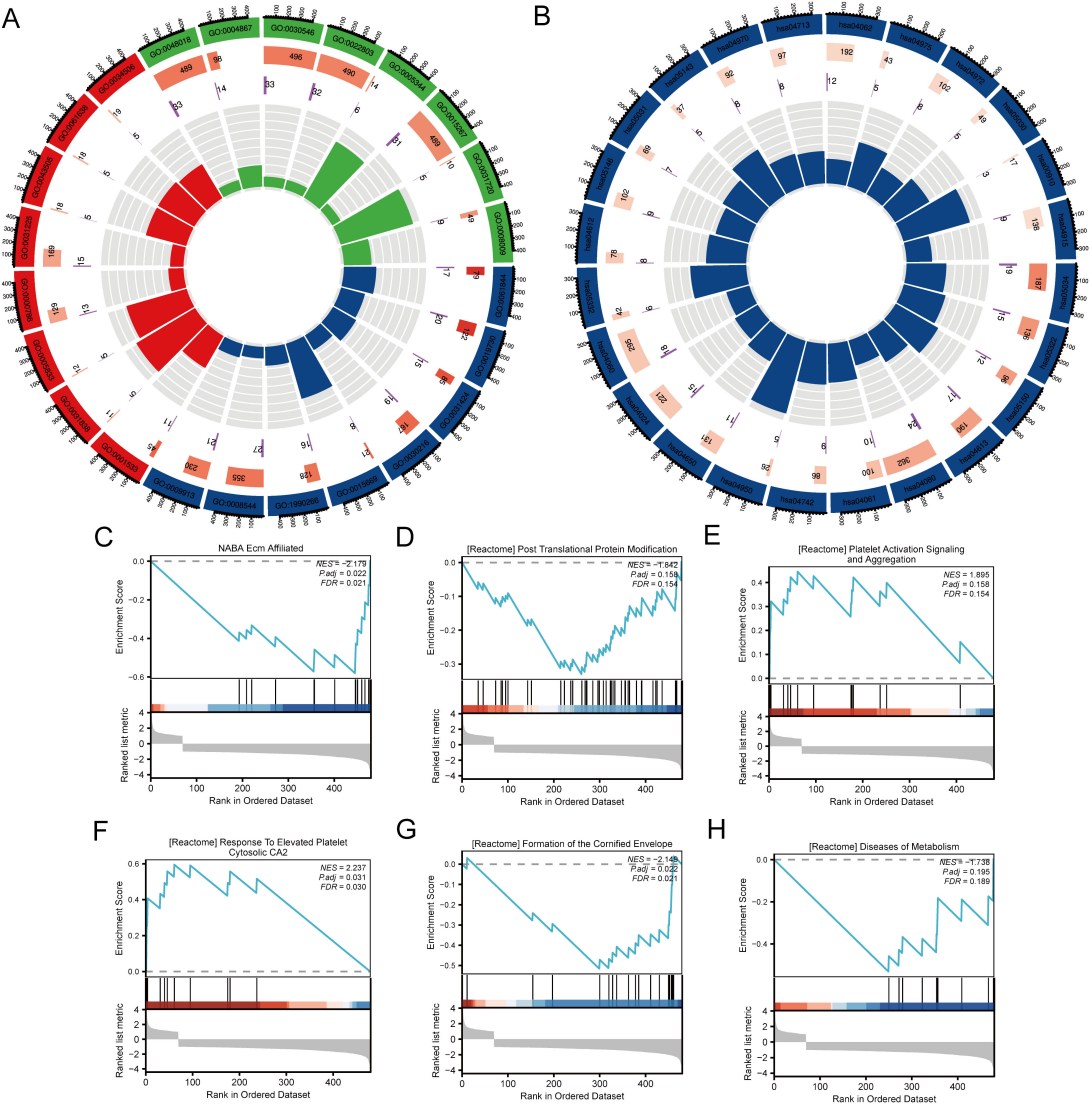
Functional enrichment analysis of SLC22A3 in colorectal cancer. **(A)** GO enrichment analysis for differentially expressed genes associated with SLC22A3. **(B)** KEGG pathway enrichment analysis for differentially expressed genes associated with SLC22A3. **(C)** GSEA enrichment plot for NABA Ecm Affiliated (NES = -2.179, Padj = 0.022, FDR = 0.021). **(D)** GSEA enrichment plot for reactome post translational protein modification (NES = -1.842, Padj = 0.158, FDR = 0.154). **(E)** GSEA enrichment plot for reactome platelet activation signaling and aggregation(NES = 1.895, Padj = 0.158, FDR = 0.154). **(F)** GSEA enrichment plot for reactome response to elevated platelet cytosolic CA2 (NES = 2.237, Padj = 0.031, FDR = 0.030). **(G)** GSEA enrichment plot for reactome formation of the cornified envelope (NES = -2.148, Padj = 0.022, FDR = 0.031). **(H)** GSEA enrichment plot for reactome diseases of metabolism (NES = -1.738, Padj = 0.195, FDR = 0.189).

This study investigates the differential expression of the SLC22A3 gene in colorectal cancer (CRC) and its associated biological pathways, utilizing Gene Ontology (GO), Kyoto Encyclopedia of Genes and Genomes (KEGG), and Gene Set Enrichment Analysis (GSEA). The analysis begins by categorizing CRC samples into high and low SLC22A3 expression groups and examining the differences in gene expression between these groups. The GO analysis elucidates the functional implications of the differentially expressed genes (DEGs) across biological processes, cellular components, and molecular functions. The results indicate significant enrichment in processes related to immune response, cell cycle regulation, apoptosis, and metabolic processes, suggesting that variations in SLC22A3 expression may impact critical cellular activities that contribute to CRC progression (**Figure 9A**). This comprehensive approach to GO analysis highlights the broad spectrum of biological activities influenced by SLC22A3, providing a foundational understanding of its role in CRC biology. KEGG pathway analysis further clarifies the biological pathways associated with SLC22A3 expression. The analysis reveals significant enrichment in pathways involved in cancer-related processes, including the PI3K-Akt signaling pathway, cell adhesion molecules, and cytokine-cytokine receptor interaction (**Figure 9B**). These pathways are integral to tumor growth, metastasis, and the tumor microenvironment, implying that SLC22A3 may modulate these processes in CRC. This pathway-focused analysis offers a detailed view of the molecular mechanisms through which SLC22A3 influences CRC pathogenesis. The study also incorporates six GSEA analyses, identifying the top enriched pathways in the context of high versus low SLC22A3 expression. These pathways provide insights into the broader biological functions and mechanisms associated with SLC22A3. The GSEA results reveal significant enrichment in pathways related to immune regulation, DNA repair, metabolic reprogramming, and signal transduction (**Figures 9C–H**). Specifically, GSEA analyses show significant enrichment in pathways such as NABA Ecm Affiliated, Reactome Post Translational Protein Modification, Reactome Platelet Activation Signaling and Aggregation, Reactome Response to Elevated Platelet Cytosolic CA2, Reactome Formation of the Cornified Envelope, and Reactome Diseases of Metabolism. These findings underscore the multifaceted role of SLC22A3 in CRC biology, suggesting that SLC22A3 may influence a diverse range of cellular and molecular processes.

In summary, this comprehensive analysis of SLC22A3 expression in CRC, integrating GO, KEGG, and GSEA, offers valuable insights into the gene’s functional roles and its potential impact on key biological processes and pathways. The findings underscore the significance of SLC22A3 as a potential biomarker and therapeutic target in colorectal cancer. The integration of multiple enrichment analyses provides a robust framework for understanding the complex biological interactions involving SLC22A3, paving the way for future research and targeted therapeutic strategies in CRC.

### Comprehensive examination of immune cell infiltration and SLC22A3 expression in colorectal cancer

**Figure 10:**
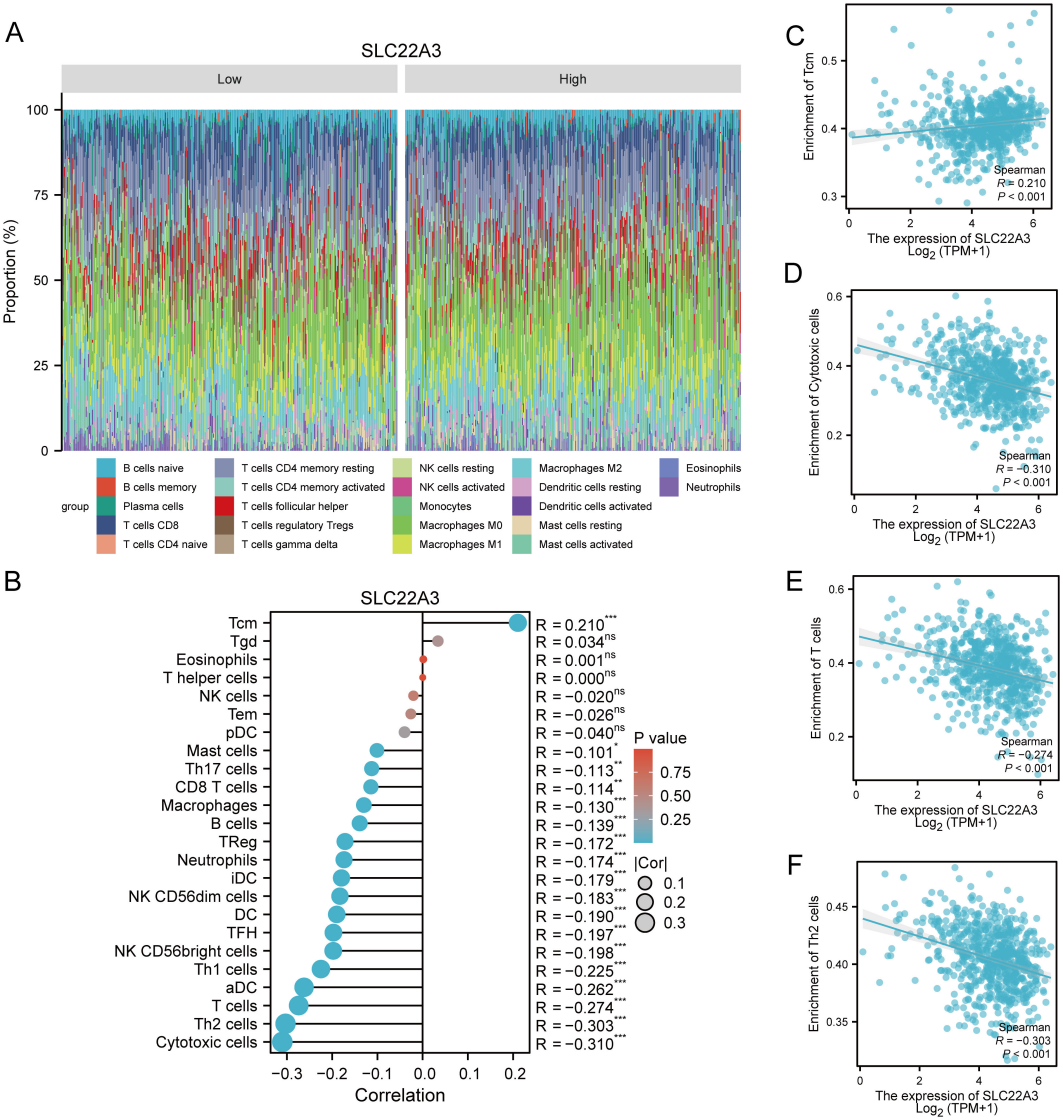
Comprehensive analysis of immune infiltration and SLC22A3 expression in colorectal cancer. **(A)** Immune cell proportion in low and high SLC22A3 expression groups. **(B)** Correlation between SLC22A3 expression and immune cell infiltration in colorectal cancer. **(C)** Correlation between SLC22A3 expression and enrichment of Tcm cells. **(D)** Correlation between SLC22A3 expression and enrichment of cytotoxic cells. **(E)** Correlation between SLC22A3 expression and enrichment of T cells. **(F)** Correlation between SLC22A3 expression and enrichment of Th2 cells.

This study provides a comprehensive examination of immune cell infiltration and its correlation with SLC22A3 expression in colorectal cancer (CRC), utilizing multiple sophisticated analytical approaches. It begins with a CIBERSORT analysis presented as a stacked bar plot, quantifying the proportions of various immune cell types within the tumor microenvironment of CRC samples. Each bar represents an individual sample, with different colors denoting distinct immune cell populations such as T cells, B cells, and macrophages, highlighting the immune landscape heterogeneity among CRC patients (**Figure 10A**). Next, a single-sample Gene Set Enrichment Analysis (ssGSEA) lollipop plot highlights the enrichment scores of various immune-related gene sets in CRC samples. Each lollipop represents an immune-related gene set, with the stick’s length indicating the enrichment score, providing a detailed view of the relative abundance and activity of different immune pathways in CRC (**Figure 10B**). The study also includes four scatter plots depicting the correlation between SLC22A3 expression levels and the enrichment scores of specific immune cells, displaying Spearman correlation coefficients to indicate the strength and direction of these relationships. **Figure 10C** shows the correlation between SLC22A3 expression (Log2 TPM+1) and the enrichment of T central memory (Tcm) cells, with a positive Spearman correlation coefficient (R = 0.210, P < 0.001). The correlation between SLC22A3 expression and the enrichment of cytotoxic cells indicates a significant negative correlation (R = -0.310, P < 0.001) (**Figure 10D**). The correlation between SLC22A3 expression and the enrichment of T cells also shows a negative correlation (R = -0.274, P < 0.001) (**Figure 10E**). Lastly, **Figure 10F** presents the correlation between SLC22A3 expression and the enrichment of T helper 2 (Th2) cells, with a significant negative correlation (R = -0.303, P < 0.001). These scatter plots provide insights into how SLC22A3 expression is associated with the presence and activity of specific immune cell types within the tumor microenvironment. Lastly, a Pearson correlation analysis illustrates the overall relationship between SLC22A3 expression and various immune-related metrics. This part of the study includes a matrix or heatmap showing Pearson correlation coefficients between SLC22A3 and a wide range of immune cell types and immune-related gene sets, offering potential implications for SLC22A3’s role in modulating immune responses and its relevance in cancer immunotherapy.

This integrated analysis underscores the significant role of SLC22A3 in the immune contexture of CRC, providing valuable insights into its potential interactions with the tumor microenvironment and its implications for cancer immunotherapy.

### Comprehensive correlation analysis of SLC22A3 expression with gene sets in colorectal cancer

This study investigates the correlation between SLC22A3 expression and various gene sets in colorectal cancer (CRC), employing Pearson correlation heatmaps and referencing specific gene sets from the literature. It begins with a heatmap depicting the Pearson correlation coefficients between SLC22A3 and multiple genes within six distinct gene sets. These gene sets, derived from relevant scientific literature, are associated with various biological processes and pathways pertinent to cancer, including immune response, cell cycle regulation, apoptosis, metabolic pathways, angiogenesis, and DNA repair. The heatmap provides a visual representation of the correlation strength, with colors ranging from blue (indicating negative correlations) to red (indicating positive correlations). Each cell in the heatmap represents the correlation coefficient between SLC22A3 and an individual gene, offering a detailed view of how SLC22A3 expression interacts with these genes at a molecular level. The immune response gene set includes genes involved in the regulation of immune responses, crucial for understanding the interaction between tumor cells and the immune system. The cell cycle regulation gene set comprises genes that control cell cycle progression and division, highlighting potential mechanisms through which SLC22A3 may influence tumor growth. The apoptosis-related gene set consists of genes associated with programmed cell death, providing insights into how SLC22A3 may affect cell survival and apoptosis in CRC. The metabolic pathway gene set involves genes in metabolic processes, illustrating how SLC22A3 might alter metabolic pathways within cancer cells. The angiogenesis-related gene set includes genes that regulate the formation of new blood vessels, indicating the role of SLC22A3 in promoting or inhibiting angiogenesis. The DNA repair gene set contains genes that participate in the repair of DNA damage, suggesting how SLC22A3 may impact genomic stability and repair mechanisms.

This study also illustrates the Pearson correlation coefficients between SLC22A3 expression and a set of chemokine genes in CRC samples (**Supplementary Figure SA**). Each cell represents the correlation coefficient between SLC22A3 and an individual chemokine gene, with colors ranging from blue (negative correlation) to red (positive correlation). Significant correlations are indicated with asterisks (*p < 0.05, **p < 0.01, ***p < 0.001), providing insights into how SLC22A3 expression correlates with the expression of various chemokines, which play crucial roles in immune cell recruitment and migration within the tumor microenvironment. Additionally, it depicts the Pearson correlation coefficients between SLC22A3 expression and a set of immune activation genes in CRC samples (**Supplementary Figure 3B**). The colors represent the strength and direction of the correlations, with blue indicating negative correlations and red indicating positive correlations. Significant correlations are marked with asterisks, highlighting the relationship between SLC22A3 expression and genes involved in activating the immune response, which can influence tumor immunogenicity and the effectiveness of immunotherapies. The study shows the Pearson correlation coefficients between SLC22A3 expression and a set of immunosuppressive genes in CRC samples (**Supplementary Figure 3C**). Each cell in the heatmap represents the correlation coefficient, with colors ranging from blue (negative correlation) to red (positive correlation). Significant correlations are indicated with asterisks, providing insights into how SLC22A3 expression is associated with genes that suppress immune responses, which can affect tumor escape mechanisms and resistance to immunotherapy. Furthermore, it illustrates the Pearson correlation coefficients between SLC22A3 expression and a set of major histocompatibility complex (MHC) genes in CRC samples (**Supplementary Figure 3D**). Each cell represents the correlation coefficient between SLC22A3 and an individual MHC gene, with colors ranging from blue (negative correlation) to red (positive correlation). Significant correlations are indicated with asterisks, providing insights into how SLC22A3 expression correlates with the expression of MHC genes, which are essential for antigen presentation and immune recognition. The study also depicts the Pearson correlation coefficients between SLC22A3 expression and a set of chemokine receptor genes in CRC samples (**Supplementary Figure 3E**). The colors represent the strength and direction of the correlations, with blue indicating negative correlations and red indicating positive correlations. Significant correlations are marked with asterisks, highlighting the relationship between SLC22A3 expression and genes involved in chemokine receptor signaling, which plays a crucial role in immune cell trafficking and tumor microenvironment modulation. Lastly, the study shows the Pearson correlation coefficients between SLC22A3 expression and a set of immune checkpoint genes in CRC samples (**Supplementary Figure 3F**). Each cell in the heatmap represents the correlation coefficient, with colors ranging from blue (negative correlation) to red (positive correlation). Significant correlations are indicated with asterisks, providing insights into how SLC22A3 expression is associated with genes that regulate immune checkpoints, which are critical for maintaining immune homeostasis and have implications for immunotherapy responses.

The detailed heatmap allows for the identification of specific genes within these sets that show significant positive or negative correlations with SLC22A3, providing insights into potential mechanisms through which SLC22A3 may influence CRC progression and patient outcomes. By integrating these correlation analyses with references to established gene sets, this study highlights the intricate molecular interactions involving SLC22A3 and underscores its potential role as a key player in the tumor microenvironment of CRC. The findings from this comprehensive correlation analysis may pave the way for further functional studies and the development of targeted therapeutic strategies.

### Analysis of target gene expression in CRC and normal colonic epithelial cell lines

**Figure 11:**
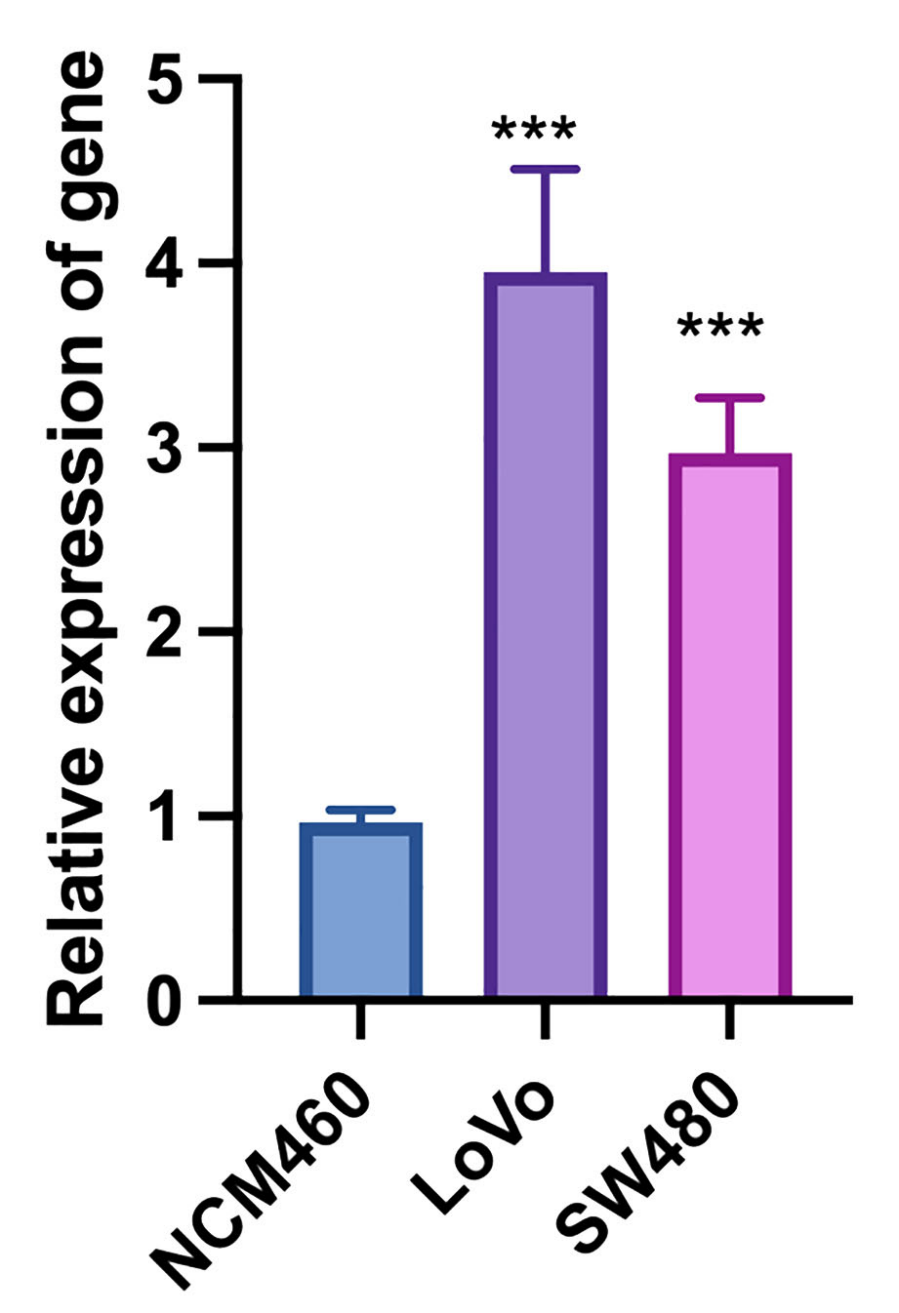
Quantitative real-time PCR analysis of target gene expression in colorectal cancer (LoVo and SW480) and normal colonic epithelial cell lines (NCM460). “***”: p < 0.001 (highly significant).

The quantitative real-time PCR results demonstrate significant differential expression of the target gene across colorectal cancer (CRC) cell lines (LoVo and SW480) and the normal colonic epithelial cell line (NCM460). The relative expression levels of the target gene were normalized to GAPDH, serving as the internal control, and analyzed using the 2^(-ΔΔCt) method (**Supplementary Table 2**). The results indicate that the target gene is markedly upregulated in both CRC cell lines compared to the normal colonic epithelial cell line. Specifically, LoVo and SW480 cell lines show a substantial increase in target gene expression, with fold changes of 4 and 3, respectively (**Figure 11**). The increased expression of the target gene in CRC cell lines suggests a potential role in the pathogenesis or progression of colorectal cancer. These findings warrant further investigation into the functional significance of the target gene in CRC and its potential as a biomarker or therapeutic target. The statistical analysis confirms the significance of the differential expression, with P-values indicating robust differences between the CRC cell lines and the normal colonic epithelial cells. Overall, the quantitative real-time PCR results highlight the importance of the target gene in colorectal cancer biology and support its further exploration in the context of cancer research and clinical applications.

## Discussion

This study provides robust evidence suggesting a causal relationship between HF and an increased risk of colorectal cancer using a bidirectional MR approach. Our findings indicate that individuals genetically predisposed to HF are more likely to develop colorectal cancer, while the reverse causal pathway does not show significant associations. These results underscore the intricate interplay between cardiovascular and oncological health, offering insights into shared pathophysiological mechanisms.

One of the key strengths of our study is the use of MR, which leverages genetic variants as IVs to infer causality. This method mitigates confounding factors and reverse causation that often plague observational studies. By employing genetic instruments, we can more reliably determine the direction and magnitude of the causal effect. The consistency of findings from various GWAS sources reinforces the robustness of the observed association between HF and colorectal cancer.

Our results align with existing literature that suggests a link between cardiovascular diseases and cancer risk (32, 33). Chronic inflammation, a hallmark of HF, is a well-known promoter of tumorigenesis. The systemic inflammatory response associated with HF may create a microenvironment conducive to cancer development. Inflammatory cytokines, such as TNF-α and IL-6, are elevated in HF and have been implicated in promoting cancer cell proliferation and survival (34, 35). Additionally, oxidative stress and the resultant DNA damage in HF patients may contribute to cancer initiation and progression (36–38). The metabolic and endocrine alterations in HF patients also provide a plausible explanation for the increased colorectal cancer risk. HF is often accompanied by insulin resistance and dysregulation of glucose metabolism, conditions that have been associated with higher cancer risk (39, 40). Hyperinsulinemia can promote cell proliferation and inhibit apoptosis, thereby facilitating cancer development (41).

Although existing studies have suggested that colorectal cancer may affect various cardiovascular functions, including left ventricular ejection fraction (42), our reverse MR study does not support this notion. Specifically, our findings do not indicate that colorectal cancer increases the risk of heart failure. The lack of association may be due to several factors. First, the genetic variants used as instruments for colorectal cancer might not capture the full spectrum of genetic predisposition, potentially underestimating the true causal effect. Second, the relatively smaller sample size for colorectal cancer compared to HF might limit the statistical power to detect a modest reverse effect. Future studies with larger cohorts and more comprehensive genetic instruments are needed to elucidate this aspect.

Our study acknowledges several limitations that warrant consideration. Despite the robustness of MR, inherent biases can affect its outcomes. Particularly, pleiotropy, where genetic variants influence multiple traits, poses a potential bias (43). Nevertheless, our sensitivity analyses, including MR-Egger and MR-PRESSO, indicate that horizontal pleiotropy is unlikely to significantly impact our findings. Additionally, the generalizability of our results may be constrained to populations of European ancestry, given that the genetic variants utilized were predominantly identified in this demographic. Replication of our study in diverse populations is essential to validate and extend our findings across different genetic backgrounds.

The potential genetic link between HF and colorectal cancer underscores the importance of comprehensive care for HF patients. Considering the increased risk observed, routine screening for colorectal cancer could be beneficial for HF patients, especially those with additional risk factors such as advanced age, family history, and specific lifestyle factors. Early detection and management of colorectal cancer in HF patients have the potential to improve overall prognosis and mitigate the burden of managing these co-existing conditions.

In conclusion, our bidirectional MR study provides compelling evidence supporting a causal relationship between HF and an increased risk of colorectal cancer. This underscores the importance of integrated approaches to managing these conditions. Shared biological pathways such as inflammation, metabolic dysregulation, and neurohormonal changes suggest potential targets for therapeutic interventions. Future research should delve deeper into these mechanisms to develop strategies for early detection and prevention in high-risk populations. By contributing to the growing body of evidence on the interplay between cardiovascular health and cancer, our study lays the foundation for future investigations into this intricate relationship.

## Data Availability

The original contributions presented in the study are included in the article/**Supplementary Material**, further inquiries can be directed to the corresponding author/s.
