# Supplementary material for: Using multi-omics to explore the genetic causal relationship between colorectal cancer and heart failure in gastrointestinal tumors
Source: Front Immunol. 2024 Sep 13;15:1454021. doi: 10.3389/fimmu.2024.1454021 (PMC11427256; doi:10.3389/fimmu.2024.1454021)
Supplement: Supplementary file 1 [file DataSheet1.docx]

**Supplementary table 1. A list of primers used in this study.**

| Gene | Forward sequence (5’ to 3’) | Reverse sequence (5’ to 3’) |
| --- | --- | --- |
| GAPDH | GGAGCGAGATCCCTCCAAAAT | GGCTGTTGTCATACTTCTCATGG |
| SLC22A3 | ATCGTCAGCGAGTTTGACCTT | ACCTGTCTGCTGCATAGCCTA |

Supplementary Figure 1.TIF

**
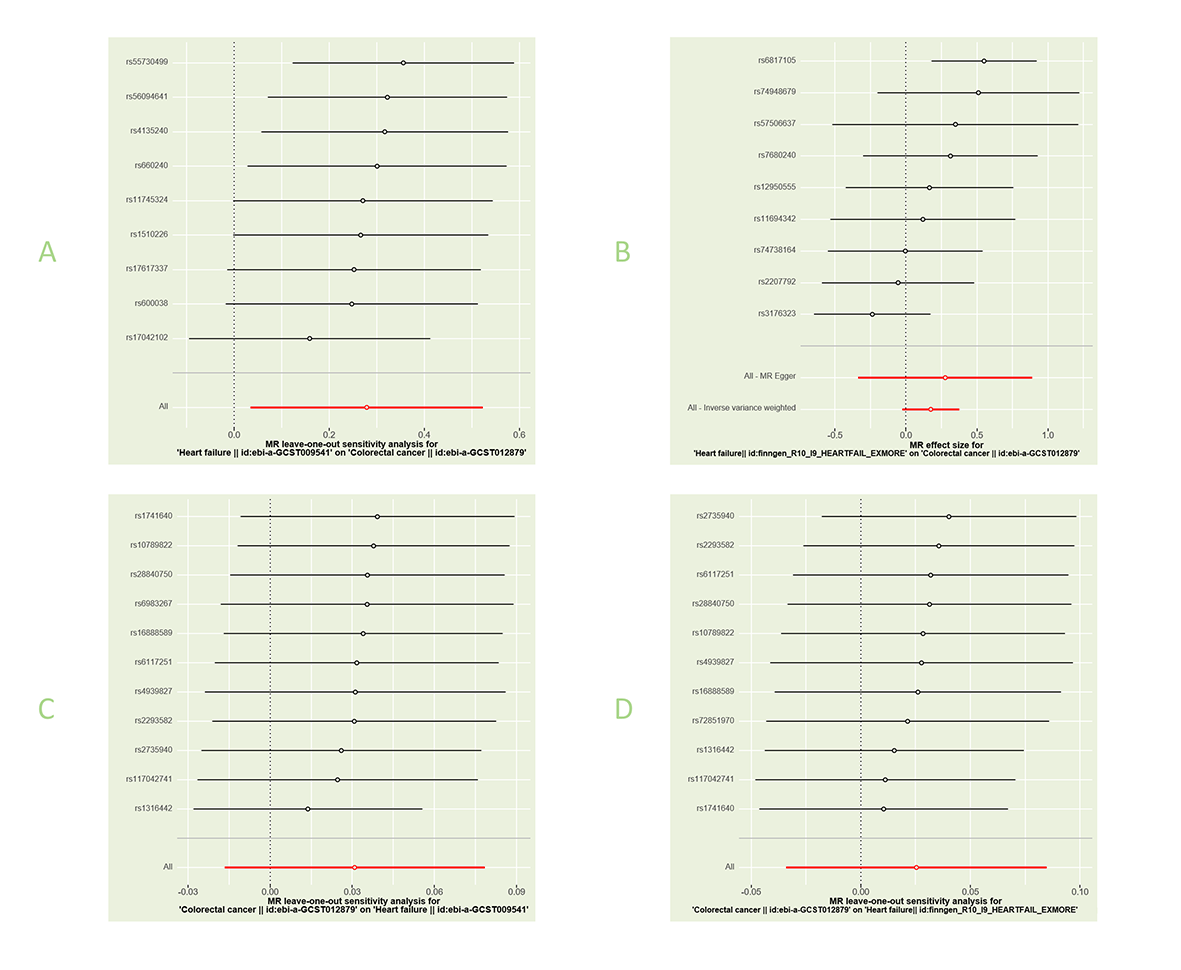
**

**Supplementary Figure 1 Results of the Mendelian Randomization (MR) analysis showing the causal relationship between a specific exposure and disease incidence.**

Supplementary Figure 2.TIF


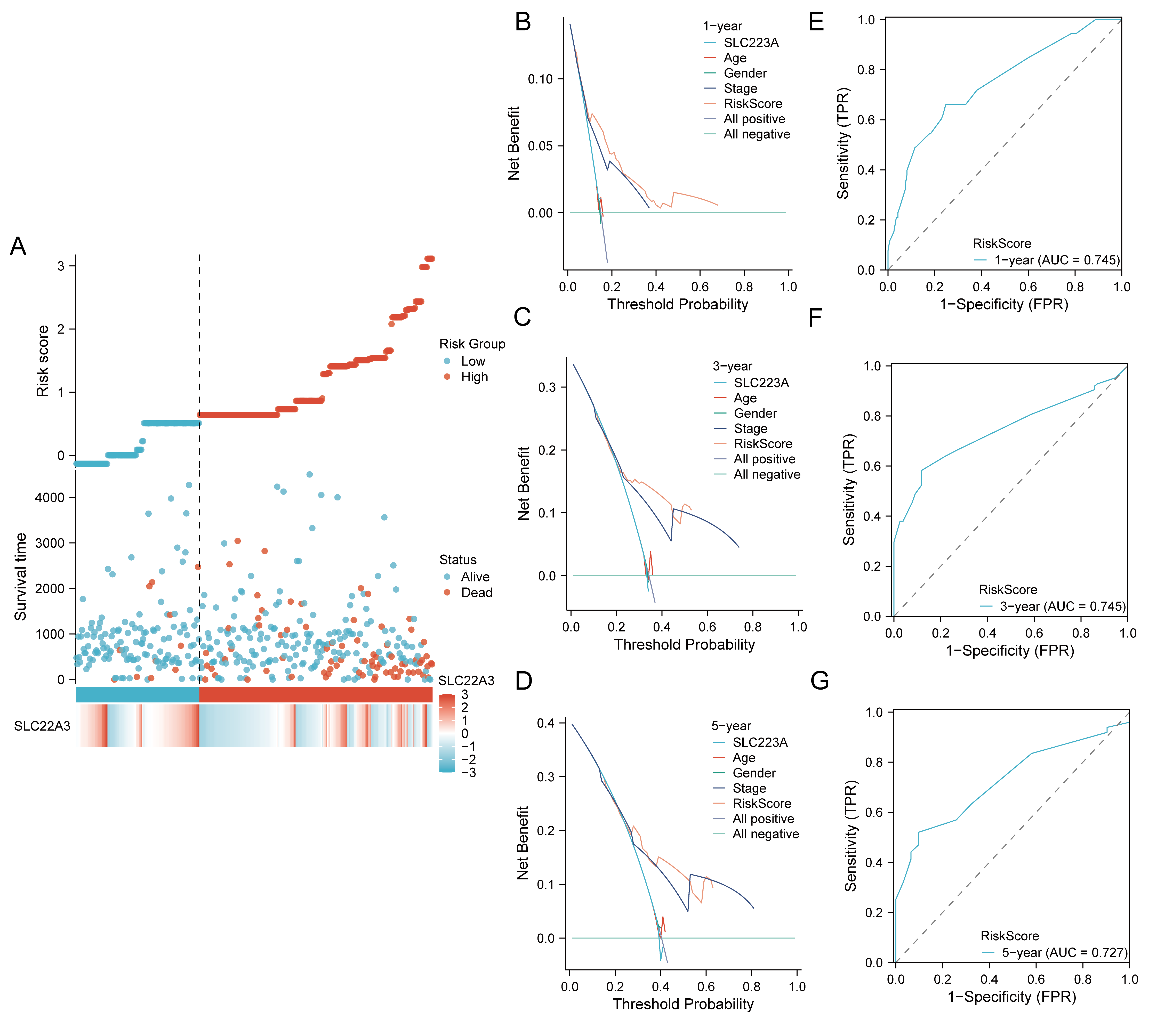


**Supplementary Figure 2 Risk Score Analysis and Survival Correlation with SLC22A3 Expression in Colorectal Cancer A**: Risk Score Distribution and Survival Analysis Based on SLC22A3 Expression. **B**: Decision Curve Analysis for 1-Year Survival Prediction. **C**: Decision Curve Analysis for 3-Year Survival Prediction. **D**: Decision Curve Analysis for 5-Year Survival Prediction. **E**: ROC Curve for 1-Year Survival Prediction. **F**: ROC Curve for 3-Year Survival Prediction. **G**:

Supplementary Figure 3.TIF


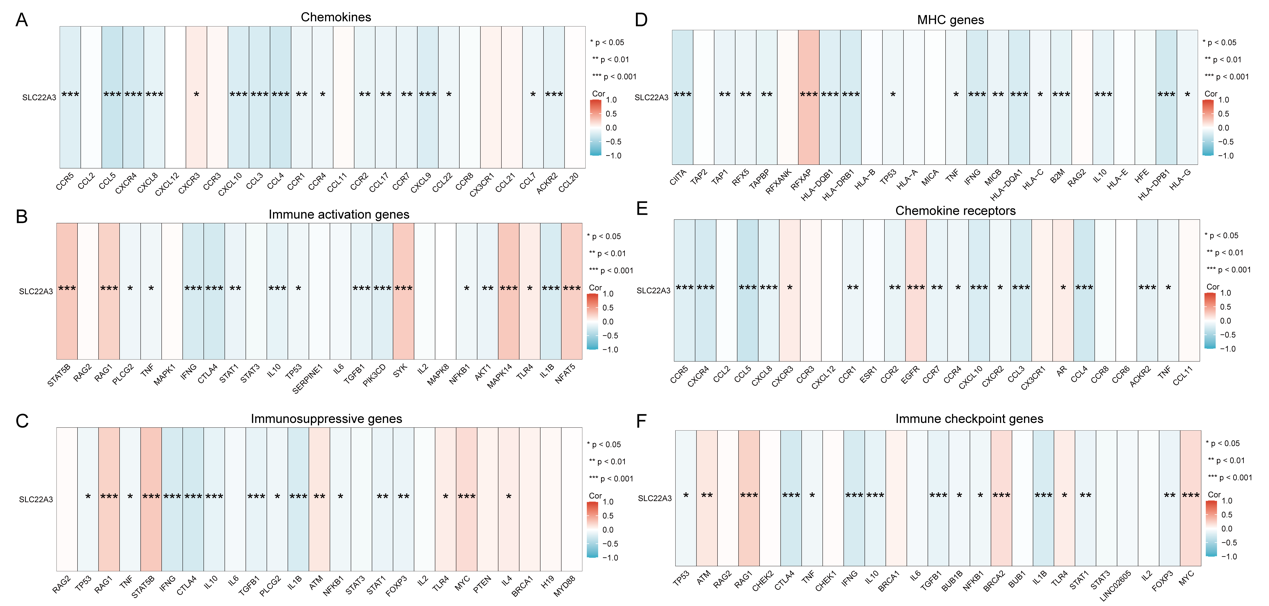


**Supplementary Figure 3 Pearson Correlation Heatmap of SLC22A3 Expression and Gene Sets in Colorectal Cancer A**: Correlation Between SLC22A3 Expression and Chemokine Genes. **B**: Correlation Between SLC22A3 Expression and Immune Activation Genes. **C**: Correlation Between SLC22A3 Expression and Immunosuppressive Genes. **D**: Correlation Between SLC22A3 Expression and MHC Genes. **E**: Correlation Between SLC22A3 Expression and Chemokine Receptor Genes. **F**: Correlation Between SLC22A3 Expression and Immune Checkpoint Genes.
